# Primary systemic amyloidosis: imaging interpretation of this complex multisystemic disease

**DOI:** 10.1259/bjrcr.20150171

**Published:** 2016-11-02

**Authors:** Indu Mitra, Ken Tung

**Affiliations:** Southampton University Hospital NHS Trust, Southampton, UK

## Abstract

This report highlights the diagnostic complexities involved in the case of a 63-year-old female who presented with a non-productive cough and shortness of breath on exertion. Initial chest radiograph demonstrated generalized abnormal interstitial lung markings with thickened peripheral septal lines. Further characterization was sought by CT scan of the chest, and given the possibility of lymphangitic carcinomatosis, a CT scan of the abdomen and pelvis was also performed. The CT scan findings revealed septal line thickening, abnormal omental soft tissue with calcified deposits and wall thickening of the stomach and proximal duodenum. A preliminary differential diagnosis of peritoneal carcinomatosis was made, but cancer markers were equivocal. A CT-guided biopsy of the “omental cake” was non-diagnostic, hence formal biopsy via laparoscopy was undertaken. While awaiting the results, the patient was readmitted with acute haematemesis. Gastric and duodenal biopsies from the endoscopic assessment were positive for Congo red stain and birefringent under polarizsed light, which was consistent with amyloidosis. Histology from the omental biopsies and additional haematological tests concurred. The patient was diagnosed with advanced systemic amyloid light-chain amyloidosis comprising diffuse pulmonary amyloidosis, calcified omental soft tissue deposits, and extensive soft tissue amyloid with cardiac and gastrointestinal involvement. We discuss the spectrum of differential diagnoses posed by the imaging findings and the difficulties faced in interpreting this complex case of systemic amyloidosis.

## Clinical presentation

A 63-year-old female, retired shop assistant, was referred to the respiratory clinic by her general practitioner *via* the “2-week wait” pathway. She had been complaining of a non-productive cough for the past 2 months with shortness of breath on exertion and weight loss over the last few years. She had recently been treated with two courses of antibiotics but with no improvement. She was an ex-smoker of 35 years with an index of 2 pack-years. There were no other significant risk factors or relevant past medical history. On examination, her oxygen saturation was 97% on room air, with a normal blood pressure and no clinical evidence of finger clubbing or peripheral oedema. Chest auscultation revealed mild bilateral crackles and good air entry.

**Figure 1.**
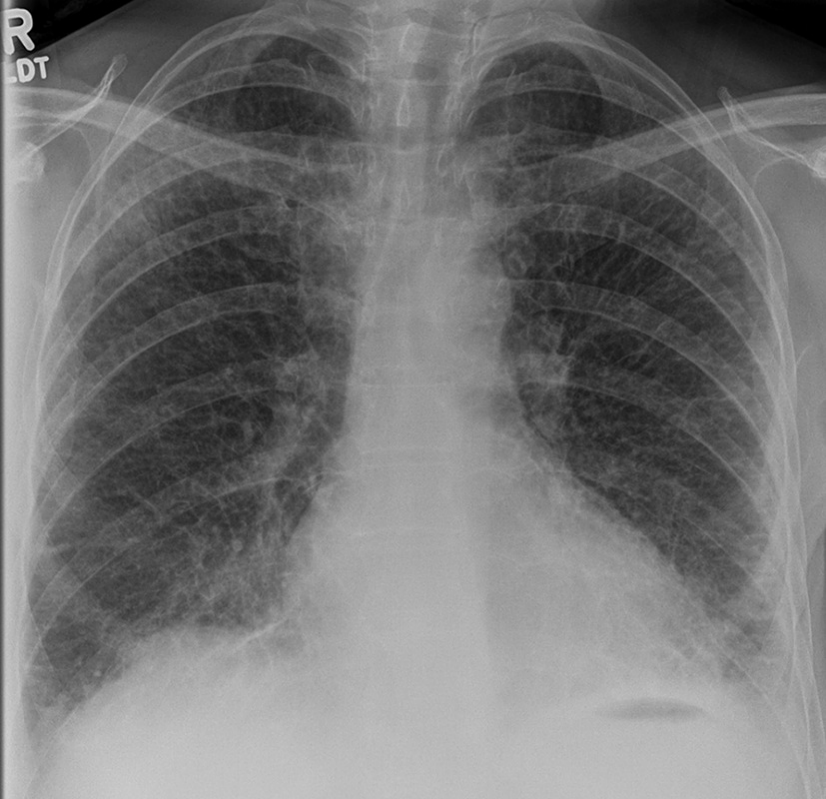
Chest radiograph. Diffuse, abnormally thickened interstitial septal lines and small bilateral pleural effusions.

Investigations included a chest radiograph (Figure 1), which showed florid generalized abnormal interstitial lung markings giving an appearance of a honeycomb lung with peripheral septal lines and associated small bilateral effusions.

## Differential diagnosis

There is a wide range of differentials based on the chest radiograph findings of thickened septal lines. These can be caused by venous pathology such as left ventricular failure, mitral stenosis or pulmonary occlusive disease. However, the heart size was not enlarged and there were no other clinical stigmata to suggest these diagnoses.

Given the “red flag” of recent weight loss, the possibility of lymphangitic carcinomatosis was raised, even though the patient was not known to have a primary neoplasm.

Other differentials for thickening of the interlobular septa include pneumoconiosis, idiopathic bronchiectasis, pulmonary haemorrhage, diffuse pulmonary lymphangiomatosis, alveolar proteinosis, alveolar microlithiasis, amyloidosis and sarcoidosis.

## Investigations/imaging findings

**Figure 2.**
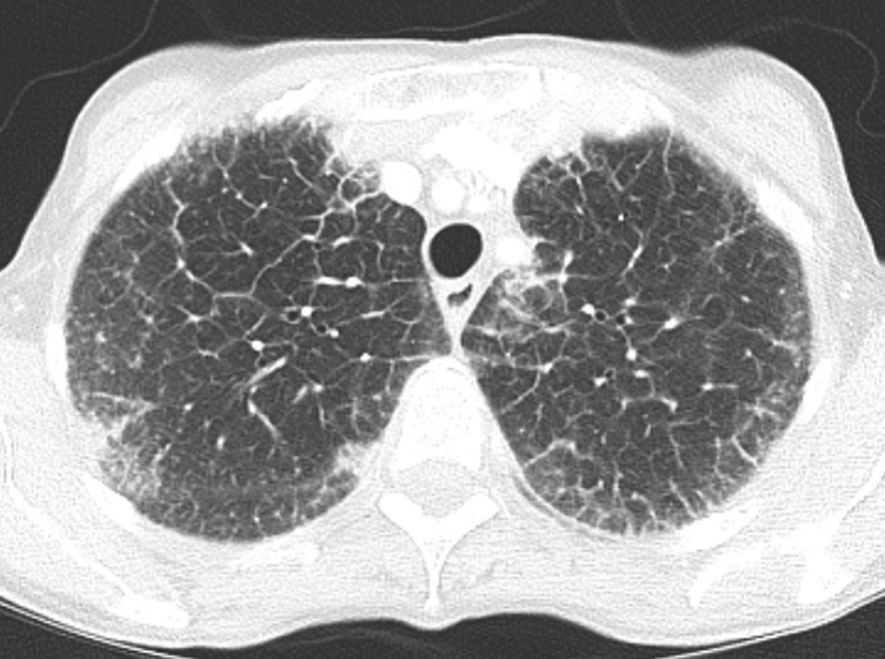
CT scan of the chest. Lung windows demonstrate thickened interstitial septal lines.

**Figure 3.**
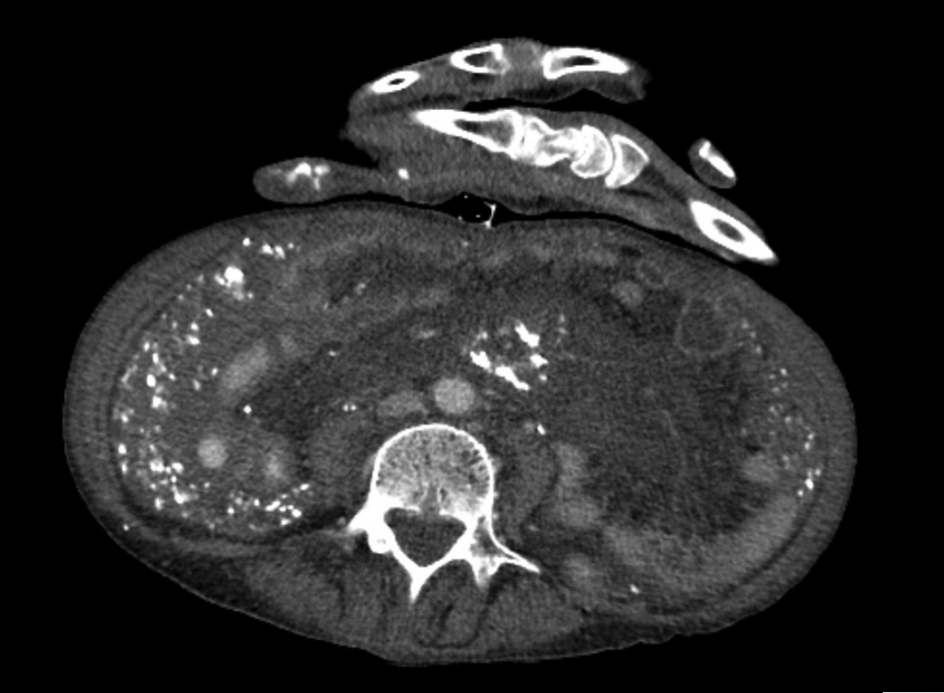
Axial CT scan of the abdomen/pelvis showing “omental cake” with calcified deposits.

**Figure 4.**
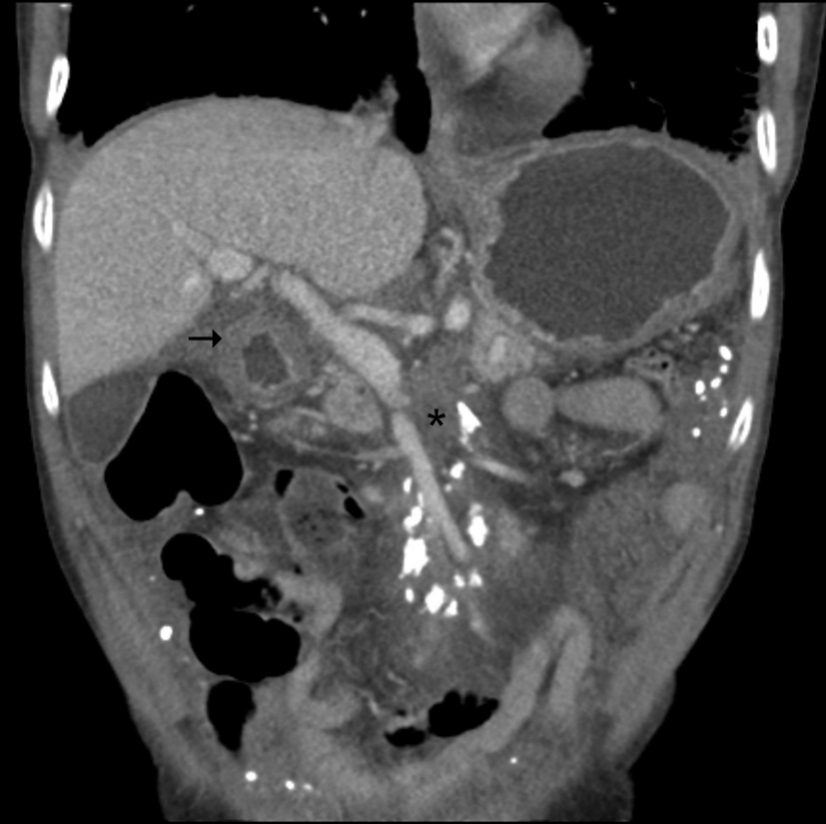
Coronal reconstruction of CT scan of the abdomen. Abnormal soft tissue with coarse calcified deposits centred by the mesenteric root (star) and a thick-walled first part of the duodenum (arrow).

Following the chest radiograph and the worrisome differential diagnosis of lymphangitic carcinomatosis, a CT scan of the chest/abdomen/pelvis was performed. The lung windows reiterated the findings of the previous radiograph, demonstrating thickened interstitial septal lines (Figure 2). The CT scan also highlighted a lesion in the left breast, marked abdominal and pelvic peritoneal thickening and “omental cake” (Figure 3). Deposits of coarse calcification were associated with the omental soft tissue, predominantly centred at the mesenteric root. Also the stomach, pylorus and first part of the duodenum appeared to have a thickened oedematous wall (Figure 4).

Based on these CT findings, several differential diagnoses were discussed. Most common causes for peritoneal disease include peritoneal carcinomatosis, pseudomyxoma peritonitis, lymphomatosis, sarcomatosis and tuberculous peritonitis.^1^ Peritoneal calcification alone can be caused by a spectrum of diseases ranging from peritoneal spread of ovarian cancer to peritoneal dialysis.^2^ Given the combination of calcification and omental soft tissue thickening, an initial presumptive diagnosis of metastatic cancer was made. The breast lesion was investigated *via* triple assessment and found to be a U2 benign lesion. Carcinoembryonic antigen and CA 125 markers were not elevated.

Histology from an urgent CT-guided biopsy of the omental thickening revealed fibrofatty connective tissue with no evidence of dysplasia or malignancy. Subsequently, formal laparoscopy and biopsy were performed.

**Figure 5.**
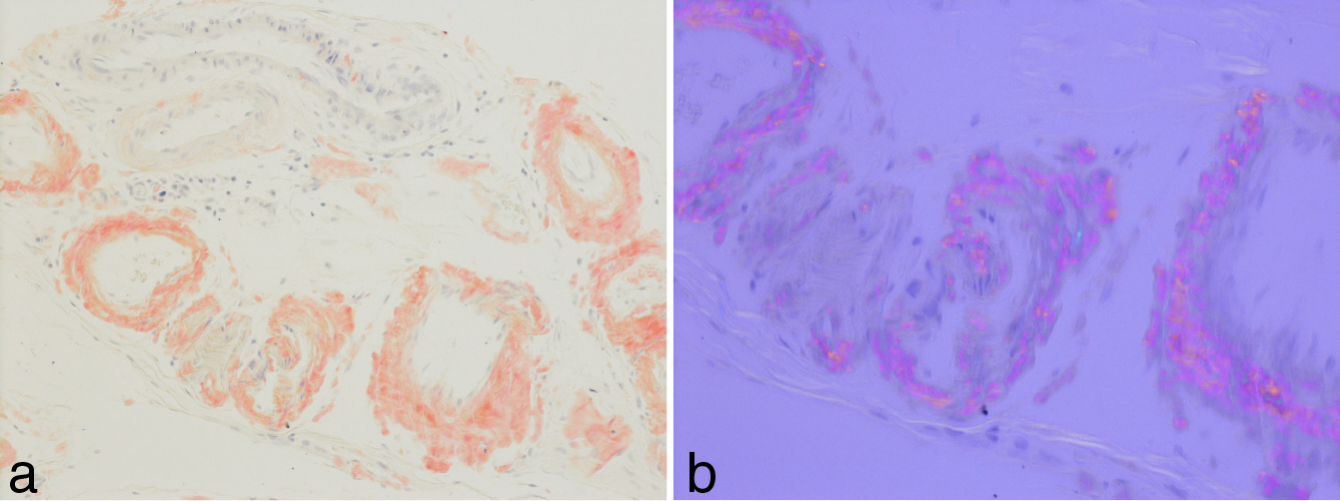
Histological images of the biopsy sample from first part of the duodenum, which are (a) positive for Congo red stain and (b) birefringent under polarized light.

Unfortunately, before histological assessment could be completed, within less than a week of the initial CT scan, the patient was admitted as an emergency presentation of haematemesis. The possibility of underlying gastrointestinal (GI) malignancy or lymphoma was raised. The patient underwent an urgent oesophagogastroduodenoscopy, which did not identify a bleeding source or mass lesion. Biopsies from the duodenal cap and proximal second part of the duodenum demonstrated fairly preserved villous architecture and a mild increase in the level of mononuclear chronic inflammatory cell infiltrate in the lamina propria. The amorphous pale eosinophilic deposits and thickened vessel walls were suspicious for amyloid. Further staining of these samples and the previous omental biopsies were positive for Congo red and birefringent when examined under polarized light (Figure 5a,b). These findings were consistent with amyloidosis.

Additional cardiac investigation by transthoracic echocardiography demonstrated a moderately dilated left atrium, moderately impaired left ventricular function and echogenic soft tissue adjacent to the left ventricle. Dynamic images showed the basal and anteroseptal regions to be hypo/akinetic. These findings were felt to be consistent with cardiac amyloidosis.

Based on the haematology, imaging and histology results, the diagnosis of advanced systemic amyloid light-chain (AL) amyloidosis was made, comprising diffuse pulmonary amyloidosis, peritoneal/omental soft tissue deposits, extensive soft tissue amyloid with GI involvement and Mayo Stage III cardiac amyloidosis.

## Treatment

The current treatment for systemic AL amyloidosis is prompt suppression of the production of amyloidogenic monoclonal immunoglobulin light chains with chemotherapy. If this is achieved, it could halt the ongoing amyloid accumulation and theoretically facilitate improvement in amyloid-related organ dysfunction and potentially lead to gradual amyloid regression.

## Outcome and follow-up

The patient began a chemotherapy regimen as per the cyclophosphamide–bortezomib–dexamethasone protocol. Depending on the dosages, each cycle of treatment can potentially carry up to 1–2% risk of death owing to toxicity. However, untreated AL amyloidosis is progressive and potentially fatal within 5 years. The success rate can vary between treatments, but, on average, approximately 40–60% of cases have a good response.^3^ Newer “targeted” therapeutics are currently being assessed in clinical trials.^4^

Imaging undoubtedly plays an important part in follow-up. Whether this be *via* cross-sectional studies or “radionuclide” imaging techniques, such as 3,3-diphosphono-1,2-propanodicarboxylic acid (DPD) or serum amyloid P component (SAP) scintigraphy, where a radionucleotide is attached to the serum amyloid component to demonstrate the amount and distribution of amyloid in the body.

With regard to this case report, despite successfully completing the first cycle of chemotherapy, the patient suffered worsening gastroparesis and heart failure owing to amyloid infiltration of the bowel and heart, and died within 7 months of presentation.

## Discussion

Systemic amyloidosis is a rare multisystemic disease with an estimated incidence in UK of 0.8/100,000 population.^5^

It can be a difficult disease to diagnose, since there is a diverse range of imaging abnormalities, which can occasionally precede haematological diagnosis. Imaging interpretation can also be complicated because the disease can affect a large variety of soft tissues and organs. Published case reviews have highlighted major abdominal solid organ involvement, such as the spleen, liver, gallbladder wall and kidneys.^6^ There are documented cases of brain deposits; nodal, muscular, synovial and ligamental infiltration; and reports of disease in the nasopharynx, orbit, lacrimal and salivary glands.^7^

In this case, the initial presentation was of pulmonary involvement. There are three different types of amyloid infiltration described: tracheobronchial deposition, diffuse parenchymal or alveolar septal involvement, and parenchymal nodules or amyloidoma.^8^ The first two types tend to have a poorer prognosis than the nodular type. Imaging features of tracheobronchial deposition are of consolidation, bronchiectasis and hyperinflation. This case report demonstrates diffuse parenchymal/alveolar septal amyloidosis infiltration with interlobular septal thickening and reticulation. Although not seen in this case, scattered micronodules can also be present.

Primary amyloidosis can often involve the cardiac tissue, which can be clinically silent or present with restrictive myopathy or cardiac failure. This case highlights the typical echocardiography findings. Other forms of imaging involve a cardiac MRI,^9^ which can demonstrate concentric biventricular myocardial hypertrophy with dilated atria and non-dilated ventricles, thickening of the intra-atrial septum and diffuse subendocardial delayed enhancement in a non-vascular distribution.^10^

GI involvement is common in systemic amyloidosis, commonly involving the stomach and small bowel, and, as in this case report, often presents with dysmotility and wall thickening. Peritoneal infiltration has been previously documented; however, to our knowledge, this case is the first case reported in the UK with both gastric/duodenal infiltration and calcified peritoneal, together with lung and cardiac, involvement.

## Learning points

1. Differential diagnoses of thickened septal lines in the lungs.
2. Differential diagnoses of abnormal peritoneal thickening with and without calcification.
3. The potentially complex multisystemic involvement of amyloidosis.
4. The most common imaging findings of this rare multisystemic disease.
5. Advances in treatment and imaging of primary systemic amyloidosis.

## Consent

This article does not contain identifying information. Informed consent was not obtained because the patient is deceased and, despite our efforts, we were unable to contact the next of kin.
